# P34 Hospital clinician knowledge, opportunity and motivation to prescribe short course antibiotic therapy for common infections

**DOI:** 10.1093/jacamr/dlae136.038

**Published:** 2024-08-23

**Authors:** Daniel Hearsey, Mike Wilcock, Mandy Slatter, Neil Powell

**Affiliations:** Pharmacy Department, Treliske Hospital, Royal Cornwall Hospitals NHS Foundation Trust (RCHT), UK; Pharmacy Department, Treliske Hospital, Royal Cornwall Hospitals NHS Foundation Trust (RCHT), UK; Pharmacy Department, Royal United Hospital Bath (RUHB), Bath, UK; Pharmacy Department, Treliske Hospital, Royal Cornwall Hospitals NHS Foundation Trust (RCHT), UK

## Abstract

**Background:**

Antibiotic use drives antimicrobial resistance (AMR), with longer durations increasing the risk of AMR and patient harm through side effects.^1,2^

**Objectives:**

We aimed to assess clinicians’ knowledge of the course length recommendations for the management of community-acquired pneumonia (CAP), hospital-acquired pneumonia (HAP), infective exacerbation of chronic obstructive pulmonary disease (IECOPD), cellulitis and pyelonephritis.^3^ In addition, we explore clinician opportunity and motivation for prescribing short course therapy.

**Methods:**

A Google forms survey was developed using the COM-B behaviour change model to explore the barriers and enablers to short antimicrobial course length prescribing. The questions were developed by the study team and piloted. The survey link was emailed to all prescribers working in adult medical specialties in two hospitals in England. Reminders were sent out periodically over a 6 week period between 29 November 2023 and 10 February 2024. Survey responses from both hospitals were pooled before analysis.

**Results:**

One hundred and sixty-five responses were completed: 82 from RCHT and 83 from RUHB, overall response rate was 23%. Overall, 124 responders were medics (75%) and 31 (19%) were from surgical specialities; 10 (6%) did not state their speciality. Knowledge of the recommended short antimicrobial course lengths was high overall with 72% of responses correctly opting for the recommended short antibiotic course lengths for each of the infections. Knowledge on short course lengths for severe CAP/HAP was an exception with only 72 (44%) respondents opting for the shorter 5 days compared with 92 (56%) for protracted courses; one respondent opted for 3 days. A quarter of respondents reported familiarity with the published evidence for short courses. Most respondents said course length recommendations were clearly indicated in the hospital’s guidelines, they were able to prescribe the courses they believed to be appropriate, and the e-prescribing system facilitated prescribing short courses. The majority didn’t believe longer courses were more effective than shorter courses, acknowledging the increased risk of AMR and side effects. Factors that contributed to protracted courses lengths were diagnostic uncertainty and slow to improve clinical signs of infection, with a minority prescribing longer courses to protect them as prescribers. Ward pharmacist challenge of protracted course lengths and audit feedback would motivate the majority to prescribe short course therapy.

**Conclusions:**

Knowledge of recommended short course therapy for the management of non-severe CAP/HAP, IECOPD, cellulitis and pyelonephritis was high but low for severe CAP/HAP. Slow to resolve symptoms and diagnostic uncertainty were identified as barriers to short courses.
